# Single-Donor and Pooling Strategies for Fecal Microbiota Transfer Product Preparation in Ulcerative Colitis: A Systematic Review and Meta-analysis

**DOI:** 10.14309/ctg.0000000000000568

**Published:** 2023-02-24

**Authors:** Benoît Levast, Mathieu Fontaine, Stéphane Nancey, Pierre Dechelotte, Joël Doré, Philippe Lehert

**Affiliations:** 1 MaaT Pharma, Lyon, France;; 2 Questel Consulting, Grenoble, France;; 3 Department of Gastroenterology, CHU de Lyon, Lyon-Sud Hospital, University Claude Bernard Lyon 1 and CIRI-INSERM U1111, Lyon, France;; 4 CHU Rouen, Rouen Normandie University INSERM 1073, Rouen, France;; 5 Université Paris-Saclay, INRAE, MetaGenoPolis, AgroParis Tech, MICALIS, 78350, Jouy-en-Josas, France;; 6 Faculty of Management, UCL, Louvain, Belgium;; 7 Faculty of Medicine, University of Melbourne, Australia.

**Keywords:** Ulcerative colitis, Fecal microbiota transfer, Pooling, Meta-analysis

## Abstract

Patients with ulcerative colitis (UC) have a less diverse microbiome than healthy subjects. Multiple studies have evaluated fecal microbiota transfer (FMT) in these patients using different methods of product preparation, doses, and routes of administration. A systematic review and meta-analysis was performed to compare the efficacy of single-donor (SDN) and multidonor (MDN) strategies for product preparation.

**METHODS:**

Systematic searches were performed in Web of Science, Scopus, PubMed, and Orbit Intelligence for studies comparing FMT products manufactured using SDN or MDN strategies to placebo in patients with UC. Fourteen controlled studies were selected for meta-analysis (10 randomized and 4 nonrandomized). The treatment response was assessed by using fixed- and random-effects models, and the significance of the indirect difference between the interventions was assessed using a network approach.

**RESULTS:**

Considering all 14 studies, MDN and SDN were superior to placebo in terms of treatment response (risk ratios [RRs]: 4.41 and 1.57, respectively [*P* ≤ 0.001 for both]), and MDN was superior to SDN (RR: 2.81, *P* = 0.005). Meta-analysis of the 10 studies with high quality of evidence showed that MDN was superior to SDN in terms of treatment response (RR: 2.31, *P* = 0.042). Results were identical for both models.

**DISCUSSION:**

There was a significant clinical benefit (remission) for patients with UC who received FMT with products manufactured by MDN strategies. Reduction of donor effect may lead to a gain in microbial diversity that could improve response to treatment. These results may have implications in the treatment approach of other diseases amenable to microbiome manipulation.
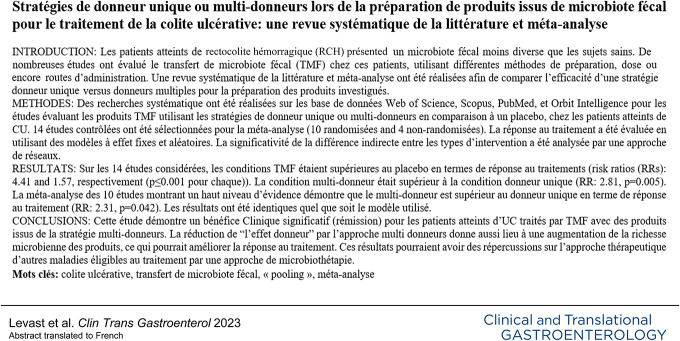

## INTRODUCTION

Ulcerative colitis (UC) is a chronic, relapsing, and remittent inflammatory disease of the colon occurring at the interface between luminal contents and the mucosal immune system. Increasing evidence implicates the colonic microbiome in the pathogenesis of UC, with microbial antigens contributing to aberrant immune activation. Patients with UC have less diverse microbiota compared with healthy subjects whatever the level of disease activity (1). This is predominantly attributable to a loss of immune-protective symbionts and increase in proinflammatory bacteria, particularly overabundant species including *Escherichia coli* and other Enterobacteriaceae (2). The usual medical therapies targeting the microbial environment (antibiotics, probiotics, and prebiotics) are not effective enough or ineffective and not recommended to induce or maintain remission (3). Fecal microbiota transfer (FMT) has been shown to be clinically effective in patients with *Clostridioides difficile* infection, where modification of the colonic ecosystem alters the disease process (4–6). Recently, FMT has been extensively studied in active UC patients in randomized controlled trials (RCTs) with various protocols. Published results from systematic reviews and meta-analysis have been largely positive (7,8), raising hope for new promising therapeutic approaches to achieve remission in active UC (9–11). The methodologies and results of these studies are not consistent, with product preparation, dosing, and route of administration representing sources of heterogeneity (7). By conceptually altering the disease process by modifying the colonic ecosystem, FMT may be expected to restore homeostasis of biochemical and antigenic drivers of immune-mediated diseases.

The objective of this systematic review with meta-analysis was to compare the efficacy of multidonor (MDN) and single-donor (SDN) strategies for FMT product preparation in achieving response to treatment of active UC. In this study, the intervention was considered to be FMT. The strategies differed in that at any individual treatment timepoint, a patient with UC was treated with a product manufactured from a single donor (SDN strategy) and from at least 2 different donors (MDN strategy).

## METHODS

The systematic review and meta-analysis were conducted as per the Preferred Reporting Items for Systematic Reviews and Meta-Analyses method (12). The protocol was registered with the International Prospective Register of Systematic Reviews (PROSPERO, registration number: CRD42020210649). The statistical analysis plan was locked before statistical analysis.

### Search strategy and selection criteria

Included studies had to report at least 1 efficacy endpoint and meet the following eligibility criteria.

1. Patients: diagnosis of UC, irrespective of follow-up duration, concomitant medication, sex, age, or language; these variables were recorded and used as meta-regressive moderators;
2. Interventions: considered to be microbiotherapy based on an ecological and complete ecosystem such as FMT with product administered at any individual treatment timepoint produced by SDN/MDN strategies;
3. Comparators: placebo (control treatment):
  - Autologous FMT: patient microbiota reconstituted at the treatment timepoint from patient's own stool donation at study inclusion. This perfectly controls the process and identifies the benefit of exogenous microbiota. Changes in patient's gut physiology between donation and treatment timepoint could introduce bias, in which case the autologous treatment could be beneficial, have no impact, or have a negative impact on the patient;
  - Saline buffer control: saline buffer administered into the patient gut to control the enema process is the best inert control compared to heterologous FMT. The quality of the blinding process is not as good for saline buffer, but no activity bias is expected.

Two investigators independently searched the current literature for articles, books, and abstracts related to the efficacy and safety of microbiota-derived drugs, irrespective of language, and checked selected references manually. The investigators searched scientific articles on Scopus, PubMed, Web of Science, and Registers, and patents on Orbit Intelligence, for documents containing clinical data assessing FMT in inflammatory bowel disease, and identified records of interest by searching websites of organizations and citation searching (see Supplemental Digital Content 1, http://links.lww.com/CTG/A910). The last search was completed on 28 June 2022.

**Figure 1.**
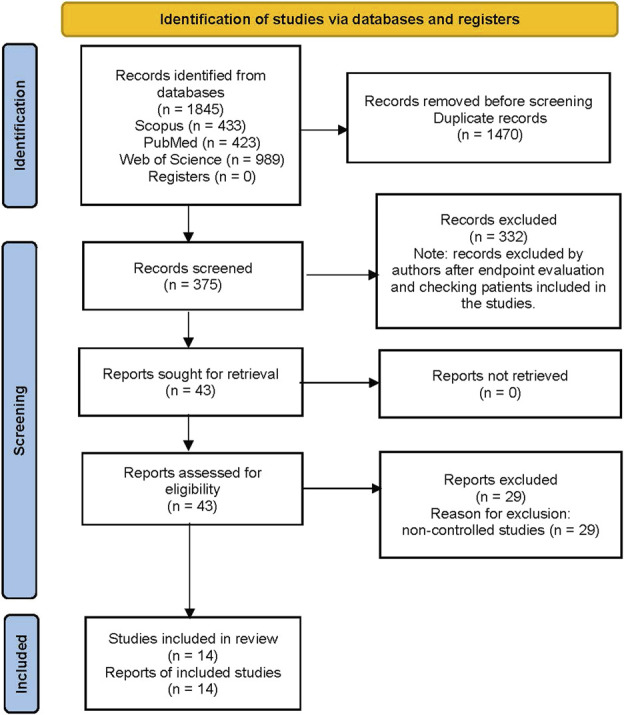
PRISMA flow diagram showing the study selection process. n, number of records; PRISMA, Preferred Reporting Items for Systematic Reviews and Meta-Analyses. From Page et al. (12).

Two investigators (B.L. and M.F.) independently assessed abstracts of selected references for eligibility; any disagreement was resolved by a third investigator (P.L.). Eventually, studies reporting on use of FMT in patients with UC were selected. If several articles reported results of the same clinical trials, the article reporting the most extensive information was selected. Potentially relevant articles were evaluated in more detail using predesigned forms to assess eligibility independently, according to predefined criteria. Studies were excluded after endpoint evaluation and because included patients did not meet the eligibility criteria for this analysis. Selected records were retrieved and further assessed for eligibility. The selection process is summarized in Figure 1 (12).

**Figure 2.**
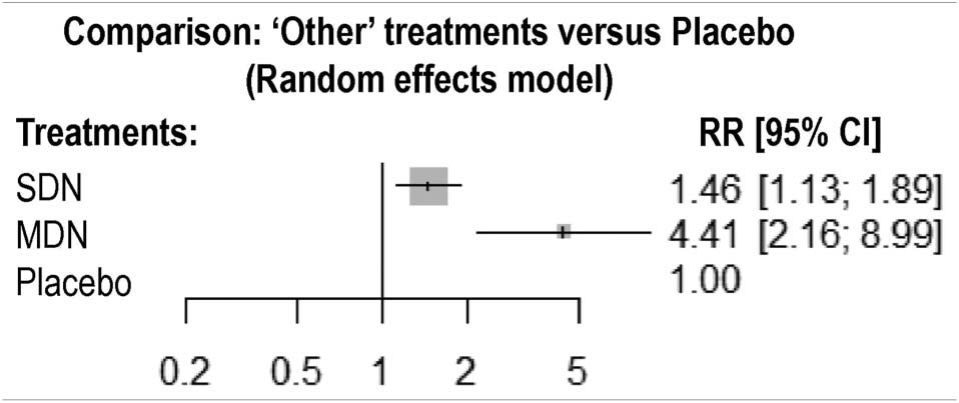
Forest plot. CI, confidence interval; MDN, multidonor; SDN, single donor; RR, risk ratio. Comparison of SDN and MDN was performed by using placebo as the null reference. RR SDN/placebo and MDN/placebo.

### Study selection

B.L. and P.L. first read the Material and Methods sections of each selected article to decide whether the study met the eligibility criteria for the meta-analysis and reached a consensus through face-to-face discussion. Subsequently, the authors attempted to identify articles based on the same raw data. The reasons for excluding studies were summarized and documented. Finally, only studies with a control arm were included in the review and meta-analysis.

### Data collection

All data from publications were systematically reviewed. All authors evaluated each publication. Several tables were constructed including a summary table of study characteristics (author, publication date, interventions, study design, and endpoints) (Table 1). After discussion between authors and preliminary reviews, a list of endpoints and moderators was set up. Each author built a data matrix containing results reported for the planned endpoints; these were compared and reconciled.

**Table 1.** Study characteristics

Study	Country	Year	Design	Mayo12	Antibiotic^a^	PEG^b^	Duration^c^	FMT^d^	Notes^e^	Route^f^
Paramsothy et al. (13)	Australia	2017	RCT	8	0	0	8	40	2	1
Moayyedi et al. (5)	Australia	2015	RCT	6.4	0	0	7	6	4	1
Costello et al. (14)	Australia	2019	RCT	7	0	1	8	3	2	1
Sood et al. (15)	India	2019	RCT	6	0	1	48	7	4	1
Rossen et al. (6)	Netherlands	2015	RCT	6	0	0	12	2	4	2
Crothers et al. (16)	United States	2021	RCT	6.3/6.7	1	0	12	84		1 + 3
Haifer et al. (17)	Australia	2021	RCT	5/7	1	0	8	56		3
Pai et al. (18)	Canada	2021	RCT	PUCAI	0	0	6	12		1
Březina et al. (19)	Czech Republic	2021	RCT	6	0	0	6	10		1
Sarbagili Shabat et al. (20)	Italy and Israel	2022	RCT	6 (SCCAI)	0	1	2	3		1
Subhadra (21)	United States	2016	CC	8	0	0	7	8		3
Kump et al. (22)	Austria	2017	CC	8	1	1	13	5		1
Scaldaferri et al. (23)	Italy	2015	CC	6.4	0	0	12	3	3	1
Ishikawa et al. (24)	Japan	2019	CC	8	1	0	4	1	3, 4	1
Borody et al. (4)	Australia	2003	C	6.4	1	1	6	5	4	1
Angelberger et al. (1)	Austria	2013	C	7.3	1	0	12	3	4	2
Kump et al. (25)	Austria	2013	C	8.9	0	0	12	1	4	1
Kunde et al. (26)	United States	2013	C	P	0	0	4	5		1
Ren et al. (27)	China	2015	C	7.3	0	0	4–30	1	4	2
Suskind et al. (28)	United States	2015	C	P	1	0	12	1		2
Damman et al. (29)	United States	2015	C	6.2	0	0	12	1	4	1
Wei et al. (30)	China	2016	C	5.8	1	1	12	1		1
Vermeire et al. (31)	Belgium	2016	C	8	0	0	8	2	4	2
Karakan et al. (32)	Turkey	2016	C	-	0	1	12	1–6		1
Mizuno et al. (33)	Japan	2017	C	8	0	1	12	1	4	1
Nishida et al. (34)	Japan	2017	C	6	0	1	8	1		1
Jacob et al. (35)	United States	2017	C	7.5	0	1	4	1	2	1
Uygun et al. (36)	Turkey	2017	C	10	0	0	12	1	4	1
Karolewska-Bochenek et al. (37)	Poland	2017	C	PUCAI	0	0	4	8		2
Goyal et al. (38)	United States	2018	C	PUCAI	0	0	26	1		2
Tian et al. (39)	China	2019	C	5	0	1	18	5	1	2
Steube et al. (40)	Germany	2019	C	8.3	1	0	12	600	1, 2	3
Cold et al. (41)	Denmark	2019	C	7	0	0	12	1,250	2, 4	3
Ding et al. (42)	China	2019	C	10.3	0	0	12	1–3	2, 4	2
Adler et al. (43)	United States	2019	C	8	0	0	6	60		3
Sood et al. (44)	India	2019	C	8.9	0	0	14	7		1
Chen et al. (45)	China	2020	C	5.9	0	1	12	3		1
Chen et al. (46)	China	2020	C	10.2	0	1	-	3		-
Dang et al. (47)	China	2020	C	-	0	0	-	1		1
Ren et al. (48)	China	2021	C	9.6	0	1	18	2		1
Seth and Jain (49)	India	2022	C	6.4	0	1	12	3		1
Smith et al. (50)	United States	2022	C	6.5	0	0	6	6		3
Zhang et al. (51)	China	2022	C	7.0	0	1	6	1		1

5-ASA, 5-aminosalicylic acid; AFM, amoxycillin + fosfomycin + metronidazole; C, cohort; CC, controlled cohort; FMT, fecal microbiota transfer; Mayo12, Mayo Score (range 0–12) at baseline; MDN, multidonor; PEG, polyethylene glycol (bowel preparation); PUCAI, Pediatric Ulcerative Colitis Activity Index; RCT, randomized controlled trial; SCCAI, Simple Clinical Colitis Activity Index; SDN, single donor; ST, standard therapy; Trt1, active FMT treatment group; Trt2, control treatment group (autologous product used only in the study conducted by Rossen et al. (6)); TRT, number of donors per FMT product (1 = SDN, >1 = MDN).

a Number of responders and total number of patients reported on the left and right columns, respectively.

b Antibiotic/PEG used: 0 (none); 1 (1 or more).

c Duration of follow-up in weeks.

d Number of FMT administrations during the study.

e 1: Mayo score replace by the probability of score <2 (considered as relief). Pnorm(2,mean,sd)*n where n = number of patients. 2: Studied treatment where >1 donor is considered as MDN; 3: AFM assimilated with placebo ST, standard therapy; 4: Baseline value was used. When not available, estimation based on protocol selection calculated as the truncated mean of distribution assumed to be N(6,2) following the expression sum(dnorm(q:12,6,2)*q:12)/sum(dnorm(q:12,6,2)), where M and m are the minimum and maximum values in the selection. z<-seq(m,M,.01); sum(dnorm(z,6,2)*z)/sum(dnorm(z,6,2)).

f 1 = Lower route; 2 = upper route; 3 = capsules.

Data were extracted using a predetermined computer data entry template including author, publication year, trial start date, countries where the trial was conducted, treatment group, sample sizes (randomized/analyzed, by group), inclusion/exclusion criteria, mode of administration, dose and duration of treatments, outcomes, and source of funding.

Collected data were

1. Proportions originating from categorical or binary variable requiring the category count and the group sample size;
2. For continuous data, mean (SD) or median (interquartile range), and sample size by group.

### Outcome assessment

After preliminary examination of eligible studies, the investigation was limited to the main endpoint, concentrating on the therapeutic response as a success/failure binary endpoint as reported by the investigator (remission in most cases). Very few other outcomes were consistently reported in the studies.

### Data availability

All data generated/analyzed during this study are included in this published article and its supplementary information.

### Meta-analysis

All studies were analyzed for certainty of evidence based on the Grading of Recommendations, Assessment, Development, and Evaluations (GRADE) approach (52). The Cochrane Collaboration's risk-of-bias tool (53) was used to evaluate bias and filled in by 3 reviewers on the 14 controlled studies (Table 2). Each study was separately analyzed for risk of bias or indirectness. Heterogeneity (using a random model), imprecision, and publication bias (using funnel plots) were evaluated at the meta-analysis level. Meta-regression was performed using evidence level as moderator.

**Table 2.** Summary of certainty of evidence including risk of bias and directness for each study

Study	Bias risk					Directness					ΣBD
	Blinding	ITT	Selected	Design	ΣBR	P	I	C	O	ΣD	
Rossen et al. (6)	+	+	+	+	0	+	+	+	+	0	H
Moayyedi et al. (5)	+	+	+	+	−1	+	−	+	+	0	H
Scaldaferri et al. (23)	−	+	+	+	−1	+	+	+	+	−1	M
Kump et al. (22)	−	+	−	−	−1	+	+	+	+	−1	M
Ishikawa et al. (24)	−	−	+	+	−1	+	+	+	+	−1	M
Sood et al. (15)	+	+	+	−	−1	+	+	+	+	−1	H
Subhadra (21)	−	+	−	−	0	+	+	+	+	0	M
Paramsothy et al. (13)	+	−	+	+	0	+	−	+	+	0	H
Costello et al. (14)	+	+	+	+	0	+	+	+	+	0	H
Crothers et al. (16)	+	+	+	−	−1	+	+	+	-	−1	M
Haifer et al. (17)	+	+	+	+	0	+	+	+	+	0	H
Pai et al. (18)	+	+	+	−	−1	+	+	+	-	−1	M
Březina et al. (19)	−	+	+	−	−1	+	+	+	+	0	M
Sarbagili Shabat et al. (20)	−	+	+	+	−1	+	−	+	-	−1	M
Heterogeneity	*I*^2^ = 0%	A nonsignificant heterogeneity was demonstrated									+
Precision	OIS = 492	Sample size (656) exceeds the OIS, and all results were significant									+
Publication bias	*P* = 0.51	No asymmetry was observed on the funnel plots									+
Total	Results with high level of certainty of evidence considered										

Funnel plot is provided in Supplemental Figure 1 (see Supplemental Digital Content 2, http://links.lww.com/CTG/A911).

∑BR, summary of bias risk; ∑BD, summary of bias risk and directness; ∑D, summary of directness; H, high certainty of evidence; ITT, intention to treat; M, moderate certainty of evidence; n, number of patients; OIS, optimal information size; PICO, patient selection, intervention, control, and outcomes.

The significance of the indirect estimate of the difference between MDN and SDN treatments was sought through a network approach (54). The risk ratio (RR) was calculated as the main calculation of effect size. A random-effects model was assumed to be most likely where difference may be expected among studies, and the fixed model was performed for sensitivity purposes. All results were compared with an alternative fixed statistical model, and heterogeneity tests were used. Correlated pairwise comparisons in multiarm studies were corrected by the weight reduction approach (55). Model fit was assessed by generalized Cochrane Qt (56,57). Treatment ranking by *P*-scores measured the extent of certainty that any one treatment was better than another, averaged over all competing treatments (58). Statistical analyses were performed using R statistical packages (version 3.2.4) and the meta-library Netmeta (59).

Data values provided as SEM were converted into SDs as per the formula SD = SEM*sqrt(n). For endpoint calculation and effect size, given the heterogeneity of the studies in their clinical definition, the following transformations were also needed for direction and measurement.

1. Severity scores (higher values meaning higher severity) were converted into improvement scores;
2. Two alternative methods were used to aggregate scales based on quantitative values or proportions:
  - Converting the proportions x/n into quantitative values in assimilating this value to a normal approximation of mean (*P* = x/n and SD = √(*P*(1-*P*))/n);
  - Conversion of quantitative difference distributed according to a normal distribution N(m,σ), assuming a success proportion of 0.5 for the tested drug.

The difference between SDN and MDN strategies was analyzed comparing all selected studies, and sensitivity analyses were performed by excluding studies with Moderate Certainty of Evidence (MCE) for each studied arm and for both arms as follows:

1. Analysis of studies with High Certainty of Evidence (HCE) for MDN and all studies of SDN;
2. Analysis of studies with HCE for SDN and all studies of MDN;
3. Analysis restricted to studies with HCE for both MDN and SDN.

The efficacy of MDN and SDN was also assessed separately vs placebo in discussing the heterogeneity and directness of the studies. Finally, meta-regressions were conducted by using the available baseline variables as potential moderators with the double purpose of assessing the confounding effect of publication date and type of study (RCT vs non-RCTs). For each covariate, a factorial model was used in testing the treatment effect, the covariate effect, and its possible interaction with the treatment. Given the low expected power of the interaction, a maximum *P* value of *P* = 0.2 was considered as significant.

## RESULTS

The search identified 1845 records in medical databases (375 records after deduplication) and 285 records from other sources (Figure 1). After exclusion of 332 studies, 43 records were retrieved and assessed for eligibility. The main criteria for further excluding studies were nonpredominance of patients with UC over those with Crohn's disease or pouchitis and case reports with fewer than 4 patients. Four pediatric studies were included (n = 48 exposed to FMT). Only 1 report identified by the patient search tool could be retrieved and assessed for eligibility (21); this was also identified in medical database search. From the 43 studies selected (29 noncontrolled studies, 10 RCTs, and 4 nonrandomized controlled studies), 14 studies with a control arm were finally selected.

Most patients in the selected studies were adults with UC; there were a few patients with Crohn's disease who were not considered in the calculation. Cumulatively, 937 patients were included in this study, of which 178 were exposed to pooled FMT products. Disease history, grading, and follow-up were heterogenous between studies but documented in this work.

### Main findings from individual studies

The outcome considered was remission. Table 1 presents study characteristics for the 43 studies examined in further detail. Results of the 14 selected studies are summarized in Table 3 Summary data are presented by intervention group (type of design and studied treatment), number of responders, sample size of each arm, percentage of responders, and benefit ratio (ratio between proportions responding to studied treatment over placebo).

**Table 3.** Selected studies

	Year	Studied treatment group^a^			Control group			Treatment^b^	TE	seTE
		Succ count	Sample size	%	Succ count	Sample size	%			
Paramsothy	2017	11	41	27	3	40	8	MDN	1.27	0.61
Moyyedi	2015	9	38	24	2	37	5	SDN	1.48	0.75
Costello	2019	12	38	32	3	35	9	MDN	1.30	0.60
Sood	2019	27	31	87	20	30	67	SDN	0.27	0.15
Rossen	2015	7	23	30	5	25	20	SDN	0.42	0.51
Subhadra	2016	16	26	62	2	24	8	MDN	2.	0.69
Kump-2	2017	4	17	24	0	10	0	SDN	1.69	1.44
Scaldaferri	2015	3	8	38	2	7	29	SDN	0.27	0.75
Ishikawa	2019	19	46	41	6	32	19	SDN	0.79	0.41
Crothers	2021	3	6	50	0	6	0	SDN	1.95	1.41
Haifer	2021	8	15	53	3	20	55	SDN	1.27	0.58
Pai	2021	11	12	92	6	12	50	SDN	0.61	0.30
Brezina	2021	12	21	57	8	22	36	SDN	0.45	0.34
Shabat	2022	4	19	21	6	15	40	SDN	−0.64	0.55

MDN, multidonor; RR, risk ratio; SDN, single donor; seTE, sE of the observed treatment effect; Succ count, count of responders; TE, observed treatment effect.

a Count of responders and sample size for the tested drug and the control groups.

b Type of medication (SDN, MDN), for each study, observed treatment effect TE (RR) and its SE (seTE).

The 14 studies included in the meta-analysis evaluated 1–84 administrations of FMT treatment over 2–48 weeks in different populations worldwide. FMT route of administration was mostly lower route, with capsules, upper route, and lower route and capsules combined in 2, 1, and 1 study, respectively. The MDN strategy was compared with placebo in 2 RCTs (13,14) which reported remission for 11/41 (27%) and 12/38 (32%) patients with FMT compared with 3/40 (8%) and 3/35 (9%) patients with placebo, respectively. The SDN strategy was compared with placebo in 8 RCTs (5,6,15–20).

### Risk of bias and indirectness within studies

There was an acceptable risk of bias for all the controlled studies; however, sensitivity analyses were required to compare results as 4 nonrandomized studies were included. The selected studies were similar to routine medical practice in terms of patient population, interventions, comparators, and outcomes, confirming that there was no indirectness within this study. External validity is synonymous with indirectness when assessing whether the data include the population, intervention, comparator, and outcome in routine medical use.

Based on risk of bias and directness, 6 studies were considered (5,6,13–15,17) as HCE and 8 studies (16,18–24) as MCE (Table 2). The Subhadra study (21) was downgraded because of lack of information for several parameters; the authors did not respond to a request for more information.

### Synthesis of results: efficacy assessment

The sample size and count of responders for each group in each study are summarized in Table 3. The 14 selected studies were HCE or MCE and compared either MDN or SDN with placebo. Both fixed- and random-effects models provided virtually the same results. The comparisons of MDN and SDN with placebo were statistically significant in favor of FMT treatments for response to therapy, with RRs (95% confidence intervals [CIs]) of 4.41 (2.14; 9.11) and 1.57 (1.23; 2.00), respectively (*P* ≤ 0.001 for both) (Table 4). The MDN strategy was shown to be significantly superior to the SDN strategy with an RR (95% CI) of 2.81 (1.38; 6.10); *P* = 0.005, with homogeneity across studies (generalized Cochran for homogeneity, *P* = 0.379, *I*^2^ = 6.6%). Finally, the *P*-scores associated with placebo, SDN, and MDN were 0.99, 0.51, and 0.02, respectively.

**Table 4.** Main results for all studies and only high certainty of evidence studies

Selection	Control	Treated	Forest plot	Statistics
ALL	PLACEBO	SDN	1.57 (1.23, 2.00)	*P* ≤ 0.001 I^2^ = 6.6% NS = 14 *χ*^2^ = 0.379 *P*-scores = 0.02, 0.51, 0.99
	PLACEBO	MDN	4.41 (2.14, 9.11)	
	SDN	MDN	2.81 (1.38, 6.10)	
HCE	PLACEBO	SDN	1.57 (1.14, 2.14)	*P* ≤ 0.001 I^2^ = 22.8% NS = 10 *χ*^2^ = 0.248 *P*-scores = 0.03, 0.48, 0.98
	PLACEBO	MDN	3.62 (1.48, 8.81)	
	SDN	MDN	2.31 (1.03, 5.85)	

Forest plots providing RR and 95% CI. Last columns (Statistics) report *P* value vs placebo for both SDN and MDN, homogeneity parameters (ratio I^2^ and *P* value of the *χ*^2^ test of homogeneity). Number of studies and *P*-scores provided for placebo, SDN, and MDM, respectively.

CI, confidence interval; HCE, high certainty of evidence; MDN, multidonor; NS, number of studies; RR, risk ratio; SDN, single donor.

By restricting the study sample to HCE studies (n = 10), MDN and SDN strategies were significantly superior to placebo in terms of response to therapy (RRs (95% CIs): 3.62 (1.48; 8.81); *P* = 0.0047 and 1.57 (1.14; 2.14), respectively; *P* = 0.0053), and MDN was superior to SDN (RR: 2.31 (1.03; 5.85); *P* = 0.042); these results were homogeneous across studies (generalized Cochran for homogeneity, *P* = 0.248, *I*^2^ = 22.8%).

Meta-regressions were conducted on available predictors at baseline (Table 5). The sensitivity of the results was first assessed with respect of nonspecific characteristics of the studies. The year of publication did not impact the treatment effect (RR = 0.96, *P* = 0.551). Likewise, RCT studies had a nonsignificant lower effect than non-RCT studies (RR = 0.66, *P* = 0.219).

**Table 5.** Meta-regression for each moderator covariate

	Effect size	95%	CI	*P* Value	Χ^2^	I^2^
Publication date (per year)	0.96	0.85,	1.09	0.551	0.319	12.70
RCT study compared with non-RCT	0.66	0.34,	1.27	0.219	0.403	3.60
Route compared with lower route						
Capsules	2.48	1.01,	6.06	0.047	0.531	0.10
Upper route	1.05	0.38,	2.91	0.931		
FMT	1.15	0.97,	1.37	0.115	0.489	0.20
Antibiotic	2.02	0.92,	2.00	0.037	0.655	0.20
Mayo score (per point)	1.05	0.57,	4.95	0.159	0.431	1.30

CI, confidence interval; FMT, fecal microbiota transfer; RCT, randomized controlled trials.

Observed effect size and 95% CI, the *P* value, and the homogeneity parameters (ratio I^2^ and *P* value of the *χ*^2^ test of homogeneity).

Baseline covariates with a potential effect on the endpoint were also evaluated. FMT delivered through capsules was associated with a higher effect (RR = 2.48, *P* = 0.047) compared with the lower route of administration of FMT; however, there was no difference overall between upper vs lower route of delivery of FMT (RR = 1.05, *P* = 0.931).

The number of FMT has a positive but nonsignificant effect (RR = 1.15 per 10 FMT, *P* = 0.115). The use of antibiotic provided a significant effect (RR = 2.02, *P* = 0.037), whereas the baseline severity Mayo index had a nonsignificant effect (RR = 1.05, *P* = 0.159). For all these meta-regressions, the effect of SDN and MDN vs placebo remained homogeneous for any variation of these baseline predictors (their interaction with treatment was invariably nonsignificant (*P* > 0.25).

## DISCUSSION

Studies of FMT in patients with active UC have assessed the effect of using single vs multiple donors for product preparation on achieving response to therapy. Patients with UC have variable clinical backgrounds, so that the “recipient effect” is more important than “donor effect” for treatment success (60,61). Pooling different donors enriches the fecal product and increases the repertoire diversity of beneficial bacteria and functionalities that could substantially improve patient's status. Richness/diversity is a surrogate marker of efficacy/clinical improvement. Product from pooled fecal material provides a full menu of bacteria that could meet the donor needs and improves chances of success for patients. This would make pooled product even more universally adapted to patients in clinical practice compared with clinical trial participants.

In studies, pooling enables product standardization and is potentially a better method of product preparation because patients of specific cohorts or subgroups could be treated with the same batch of product. This would not be possible for SDN strategies; Ren et al were unable to reproduce their previous outstanding results in patients with UC after selection of a hypothetical “FMT golden donor” (48).

Recently published systematic reviews and meta-analyses (62–66) documented the efficacy of FMT for treatment of UC. Imdad et al (66) and Liu et al (62) addressed the question of sourcing fecal samples by SDN or MDN strategies, although available data were considered insufficient to draw firm conclusions. Nevertheless, a clear consistency appeared between the published reviews and this work because similar RRs are observed in favor of FMT treatment vs control (7,63,66–68). In another clinical context, studies evaluating FMT in graft-vs-host disease (GvHD) are limited in terms of numbers of patients treated, and only SDN strategies are used for product preparation (69).

All eligible studies from this systematic review were included in the meta-analysis and restricted data to satisfactory evidence of certainty. The response to FMT therapy was consistently found to be significantly better with MDN than with SDN strategies. The network meta-analysis was straightforward, in that MDN or SDN strategies were compared with placebo, and not directly compared.

This analysis specifically looked at the potential benefit of an MDN approach in treatment of UC. Results of the main analysis showed significant difference between the strategies in favor of MDN (*P* < 0.05), confirmed by sensitivity analyses, and the magnitude of the effect size and their ratios was very similar. This benefit may be relevant to microbiome supplementation approaches in treatment of other systemic disorders and is worthy of further study.

This study demonstrates the benefit of pooling in an UC patient population where gut dysbiosis is characterized by antibiotic-related low richness, inflammation, and immunosuppression. Conditions are similar in some cancer, oncohematology, and GvHD patients. Other types of dysbiosis (e.g. ecological dysbiosis in *C. difficile* infection) might not obtain the same level of benefit from the pooling approach because correction of the lost function (colonization prevention by competitive exclusion) does involve ecological but not microbiota-host interactions and especially crosstalk with the immune system.

This study has limitations. Classification of studies was attempted on certainty of evidence. For directness in particular, there may be limitations concerning external validity for the intervention, including patient preparation before FMT (gut decontamination, antibiotics, lavage, and use of polyethylene glycol), product preparation (freshness, filtration, freezing-thawing, and lyophilization), route of administration (upper/lower, enema, colonoscopy, and duodenal/ileal area), disease severity at baseline, and additional factors such as patient's comorbidities (*C. difficile* infection). Recent meta-analyses have already reported these biases (7,8). In the selected studies, one of the main risks of bias was the use of different controls in RCTs because autologous FMT may differ from strict placebos (saline buffer/water). Nevertheless, the meta-regressions on predictors at baseline provided robust trends observed in previous studies. First, this study demonstrates a benefit of antibiotics for gut bowel preparation before FMT treatment. This is totally in line with the literature and reinforces the result and methods of this study. Secondly, capsule formulation seemed to be a game changer in the way to treat patients, providing an RR of 2.48. This probably combines two beneficial modes of action: (i) Encapsulated bacteria are slowly and consistently delivered in the small intestine to better protect the entire gastrointestinal tract. Capsules are gastro-resistant, which avoids bacteria being killed, because it could be the case for upper route administration of FMT in solution. (ii) Capsule formulation allows a drastic change in the posology, patients being treated with capsules every day and during weeks. This would certainly favor the clinical efficacy of FMT treatments.

In conclusion, this systematic review and meta-analysis synthesizes evidence in favor of the superiority of fecal products manufactured by MDN compared with SDN in FMT to achieve a clinical benefit in induction therapy in patients with active UC. Similar approaches would be of interest for other diseases (Crohn's disease or *C. difficile* infection). Reduction of donor effect may result in a gain in intestinal microbial diversity responsible for a better response to FMT. However, these findings warrant to be confirmed in a dedicated powerful RCT.

## CONFLICTS OF INTEREST

**Guarantor of the article:** Philippe Lehert, PhD.

**Specific author contributions:** All authors approved the final version of the article, including the authorship list. P.L. designed the study, and B.L. performed the literature review with the support of M.F. P.L. designed and calculated the meta-analysis, performed all statistical tasks, scored the GRADE and quality of studies, participated in the data monitoring committee, and wrote the Methods and Results sections of the publication. B.L. scored the GRADE and quality of the studies, wrote the summary of studies, introduction, and discussion, and participated in the data monitoring committee. B.L. wrote the first draft of the manuscript, and all authors contributed to the review of the data and improvement of the manuscript. Editorial support was provided by Scinopsis Medical Writing.

**Financial support:** The study was funded by MaaT Pharma, France; http://www.maatpharma.com. An unconditional grant was provided to the authors, and MaaT Pharma was not involved in writing the protocol, statistical plan, or discussion of results.

**Potential competing interests:** B.L. and M.F. are employed by the commercial company MaaT Pharma. J.D. is Scientific Advisor to MaaT Pharma. P.D. is minor shareholder of MaaT Pharma. The remaining authors declare that the research was conducted in the absence of any commercial or financial relationship that could be construed as a potential conflict of interest.

## Supplementary Material

SUPPLEMENTARY MATERIAL
