# Supplementary material for: Single-Donor and Pooling Strategies for Fecal Microbiota Transfer Product Preparation in Ulcerative Colitis: A Systematic Review and Meta-analysis
Source: Clin Transl Gastroenterol. 2023 Feb 24;14(5):e00568. doi: 10.14309/ctg.0000000000000568 (PMC10208705; doi:10.14309/ctg.0000000000000568)
Supplement: SUPPLEMENTARY MATERIAL [file ct9-14-e00568-s001.pdf]

Supplemental Digital Content – Literature Search

Article search on Web of Science (all databases)

1. TS=((F$ecal NEAR/2 Microb* NEAR/2 (administrat* OR Transplant* OR preparation$ OR formulation$ OR transfer*)) OR FMT OR (F$ecal NEAR/2 bacteriother*) OR (gut NEAR/2 microbiome NEAR/2 trans*) OR ((stool OR f$eces OR f$ecal) NEAR/2 trans*) OR (microbiome NEAR/2 therap*) OR (microbial NEAR/2 (cocktail$ OR mix$ OR blend$)))

2. TS=(((TRANSPLANT* OR TRANSFER* OR DONOR* OR ADMINIS* OR INOCU* OR DELIV* OR INFUS* OR TRANSFUS* OR *GRAFT*) NEAR/10 (STOOL$ OR F$ECAL OR F$ECES OR POOP$ OR EXCREMENT$)) AND (MICROBIOT* OR MICROBIOME* OR MICROB* OR MICROORG* OR BACTER* OR FLORA OR COMMENSAL OR CONSORT* OR MICROBIOCENOS*))

3. TS=(*donor* OR random* OR safety OR ((pool$ OR pooling OR mix$ OR mixing) NEAR/2 (STOOL$ OR F$ECAL OR F?ECES OR POOP$ OR EXCREMENT$)) OR safe OR meta-analysis OR (meta NEAR/1 analysis) OR efficacy OR effectiveness OR cohort$ OR pubmed OR embase OR scopus OR wos OR “web of science” OR CNKI)

4. TI=(pilot OR clinical OR trial$)

5. TS=(UC OR (ULCERATIVE NEAR/1 COLITIS) OR IBD OR (INFLAMMATORY NEAR/1 BOWEL NEAR/1 disease*) OR crohn* OR pouchitis OR ((INTESTINE OR BOWEL OR GUT$ OR DUODEN* OR ILEUM OR ILEOCECAL OR JEJUNUM OR JUOJENAL) NEAR/2 INFLAM*))

6. (#1 OR #2) AND (#3 OR #4) AND #5

Article search on Scopus

1. title-abs (((fecal or faecal) w/2 microb* w/2 (administrat* or transplant* or preparation* or formulation* or transfer*)) or fmt or ((fecal or faecal) w/2 bacteriother*) or (gut w/2 microbiome w/2 trans*) or ((stool or feces or faecal or fecal) w/2 trans*) or (microbiome w/2 therap*) or (microbial w/2 (cocktail* or mix or blend)))

2. title-abs (((transplant* or transfer* or donor* or adminis* or inocu* or deliv* or infus* or transfus* or *graft*) w/10 (stool or fecal or faecal or feces or faeces or poop or poops or excrement or excrements)) and (microbiot* or microbiome* or microb* or microorg* or bacter* or flora or commensal or consort* or microbiocenos*))

3. title-abs (*donor* or random* or safety or ((pool or pooling or pools or mix or mixing or mixs) w/2 (stool or fecal or faecal or faeces or feces or poop or poops or excrement or excrements)) or safe or meta-analysis or (meta pre/1 analysis) or efficacy or effectiveness or cohort* or pubmed or embase or scopus or wos or (web pre/1 of pre/1 science) or cnki)

4. title (pilot or clinical or trial*)

5. title-abs (uc or (ulcerative pre/1 colitis))

6. (#1 or #2) and (#3 or #4) and #5

Article search on PubMed

1. ((fecal[title/abstract] or faecal[title/abstract]) and microbio*[title/abstract] and (administrat*[title/abstract] or transplant*[title/abstract] or preparation*[title/abstract] or formulation*[title/abstract] or transfer*[title/abstract])) or fmt[title/abstract] or ((fecal[title/abstract] or faecal[title/abstract]) and (bacteriother*[title/abstract] or "bacterio-therap*"[title/abstract])) or (gut[title/abstract] and microbiome[title/abstract] and trans*[title/abstract])

2. (randomized[title/abstract] or randomised[title/abstract] or *donor*[title/abstract] or safe[title/abstract] or efficacy[title/abstract] or effectiveness[title/abstract] or cohort*[title/abstract] or pubmed[title/abstract] or embase[title/abstract] or scopus[title/abstract] or wos[title/abstract] or “web of science”[title/abstract] or cnki[title/abstract] or “meta-analysis”[title/abstract] or “meta analysis”[title/abstract] or safety[title/abstract] or “open-label”[title/abstract] or “open label”[title/abstract])

3. (pilot[title] or clinical[title] or trial*[title])

4. (uc[title/abstract] or “ulcerative colitis”[title/abstract])

5. #1 and (#2 or #3) and #4

Patent search on Orbit Intelligence

1. (((transplant+ or transfer+ or donor+ or adminis+ or inocu+ or deliv+ or infusion or transfus+ or +graft+) 10d (stool or fecal or faecal or f?eces or poop? or excrement?)) and (microbiot? or microbiome? or microb+ or micro_org+ or bacter+ or flora or commensal or consort+ or micro_biocenos+))/ti/ab/clms

2. (((((transplant+ or transfer+ or donor+ or adminis+ or inocu+ or deliv+ or infusion or transfus+ or +graft+) 5d (microbiot? or microbiome? or microb+ or micro_org+ or bacter+ or flora or commensal or consort+ or micro_biocenos+)) and (stool or fecal or faecal or f?eces or poop? or excrement?))/ti/ab/clms) and (intestin+ or gut? or bowel? or celiac or duoden+ or ileum or ileocecal or jejunum or juojenal or gastrointe+ or colon or rectal or colorectal or (digest+ 2d tract?))/tx)

3. (((f?ecal or microbiota or microbiome?) 3d transplant+) or (microbio* w restor* w therap*))/tx

4. (((fecal or faecal) 2d microb* 2d (administrat* or transplant* or preparation? or formulation? or composition?)) or fmt or ((fecal or faecal) 2d bacteriother*) or (microb* d restoration d therap*))/ti/ab/clms

5. ((fodder? or plant? or veget+ or fertilizer? or waste_water? or remediat+ or sewage or feed or compost+ or poultr+ or chicken?)/ti or (e03d+ or a01g+ or c02f+ or c05f+ or y02w+)/cpc/ipc)

6. ((+donor* or randomi?ed or enroll* or (pilot w stud*) or (clinical w (trial? or stud*)) or cohort* or (open w label) or safety or efficacy or ((pool or pooling or pools or mix or mixing or mixs) 2d (stool or fecal or faecal or f?eces or poop or poops or excrement or excrements)) or safe or efficacy or effectiveness) p (uc or (ulcerative w colitis)))/ti/ab/tx

7.((1 or 2 or 3 or 4) not 5) and 6
