# Supplementary figures and images for: Single-Donor and Pooling Strategies for Fecal Microbiota Transfer Product Preparation in Ulcerative Colitis: A Systematic Review and Meta-analysis
Source: Clin Transl Gastroenterol. 2023 Feb 24;14(5):e00568. doi: 10.14309/ctg.0000000000000568 (PMC10208705; doi:10.14309/ctg.0000000000000568)

Supplemental Figure 1: Funnel plot

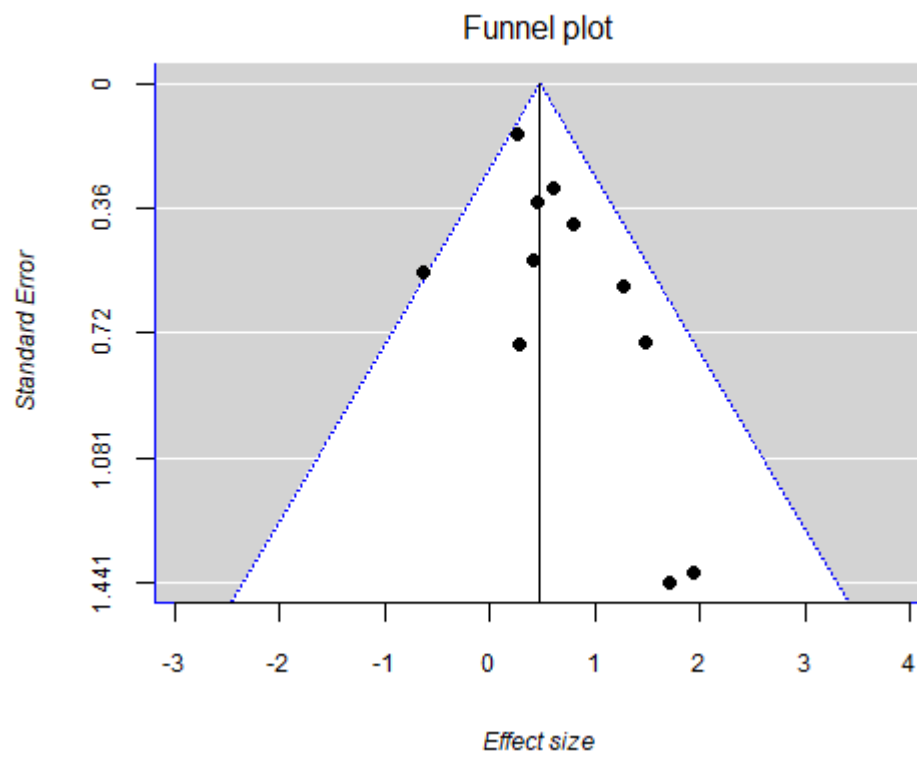

Supplement: SUPPLEMENTARY MATERIAL [file ct9-14-e00568-s002.pdf]
